# Anthropometric indices and the risk of incident sudden cardiac death among adults with and without diabetes: over 15 years of follow-up in The Tehran Lipid and Glucose Study

**DOI:** 10.1186/s13098-021-00701-z

**Published:** 2021-07-28

**Authors:** Seyyed Saeed Moazzeni, Seyed Saeed Tamehri Zadeh, Samaneh Asgari, Fereidoun Azizi, Farzad Hadaegh

**Affiliations:** 1 Prevention of Metabolic Disorders Research Center, Research Institute for Endocrine Sciences, Shahid Beheshti University of Medical Sciences, No. 24, Parvaneh Street, Velenjak, P.O. Box no19395-4763, Tehran, Iran; 2 Endocrine Research Center, Research Institute for Endocrine Sciences, Shahid Beheshti University of Medical Sciences, Tehran, Iran

**Keywords:** Sudden cardiac death, Cardiovascular disease, Anthropometric indices, Obesity, Diabetes mellitus

## Abstract

**Background:**

We investigated the association of anthropometric indices including body mass index (BMI), waist circumference (WC), waist-to-hip ratio (WHR), waist-to-height ratio (WHtR), and hip circumference (HC) with the risk of incident sudden cardiac death (SCD) among Iranian population with and without type 2 diabetes mellitus (T2DM).

**Methods:**

The study population included 9,089 subjects without and 1,185 subjects with T2DM, aged ≥ 20 years. Participants were recruited in 1999–2001 or 2001–2005, and followed for incident SCD annually, up to 20 March 2018. Multivariate Cox proportional hazard models, adjusted for traditional risk factors of cardiovascular disease, were applied to estimate hazard ratios (HRs) and 95% confidence intervals (CIs) of anthropometric indices (as continuous and categorical variables).

**Results:**

During a follow-up of over 15 years, 144 (1.58%) and 86 (7.26%) incident SCD occurred in non-T2DM and T2DM groups, respectively.

Among non-T2DM group, a 1 standard deviation (SD) increase in WHtR was associated with higher risk of incident SCD by a HR of 1.23 (95% CI: 1.00–1.50) in the multivariable model. From the first quartile to the fourth quartile of WHtR, the trend of SCD risk was significant in age- and sex-adjusted analysis (P-value for trend: 0.041). Other indices did not show significant associations with SCD.

Among T2DM group, a 1 SD increase in WHR had a HR of 1.36 (1.05–1.76) in the multivariable model. Considering WHR as categorical variables, the trend of SCD risk across quartiles of WHR was significant. Furthermore, a 1 SD increase in HC led to reduced risk of incident SCD with a HR of 0.75 (0.58–0.97) in multivariable analysis; this lower risk remained significant even after adjustment for WC. Compared to the first quartile, the fourth quartile of HC also showed a HR of 0.50 (0.25–0.99) (P-value for trend = 0.018). BMI, WC, and WHtR did not have significant associations with incident SCD.

**Conclusion:**

In our long-term population-based study, we demonstrated central but not general obesity (as assessed by WHR in participants with T2DM, and WHtR in participants without T2DM) as a herald of incident SCD. Moreover, HC can have an inverse association with SCD among participants with T2DM.

**Supplementary Information:**

The online version contains supplementary material available at 10.1186/s13098-021-00701-z.

## Introduction

Cardiovascular disease (CVD) is the cause of 18.6 million deaths in 2019 worldwide [1]. Approximately 46% of deaths and 20–23% of the burden of disease in Iran are attributed to CVD [2]. Sudden cardiac death (SCD) contributes to approximately 50% of CVD-related mortality events [3]. Owing to notable incidence, unanticipated nature, and high rate of fatality, SCD is taking into account as a public health concern [4, 5]. Coronary heart disease (CHD), with a prevalence of 7.7% among Tehranian adult residents [6], is the structural basis for approximately 70% of all SCD; however, the majority of CHD-related SCD occurs as the initial manifestation of CHD [7]; this is mainly because a great proportion of individuals experiencing SCD are not categorized into high-risk groups based on stratification of former studies [5, 7, 8]. This issue highlights the need for further investigations to identify the risk factors and high-risk groups for SCD well.

Obesity, with increasing prevalence in the world, shares common traditional cardiovascular risk factors with SCD. Traditional CVD risk factors, including hypertension, diabetes mellitus (DM), and obstructive sleep apnea, often coexist with obesity [9]. It has been firmly established that obesity maintains a mandatory role in insulin resistance (IR), a well-known risk factor for CVD [10]; however, there is an ongoing concern that which of the general or central obesity was further associated with IR [11]. Moreover, obesity was suggested to be associated with incident SCD [9, 12]; however, this association relies more on the studies considering general obesity only with a high heterogeneity [12–14]. The association of central obesity indices including waist circumference (WC) and waist-to-hip circumference (WHR) with incident SCD was examined in few researches [12, 15, 16], and no study by far investigated this association for waist-to-height ratio (WHtR) and hip circumference (HC). Furthermore, to the best of our knowledge, the association of anthropometric indices with incident SCD has not been investigated among individuals with type 2 diabetes mellitus (T2DM), who have a two-fold increase in the risk of SCD [17]. Given the considerable rise in the prevalence of overweight and obesity worldwide [18] and firm associations between obesity and several cardiovascular outcomes [19], it is noteworthy to clarify whether obesity is associated with higher risk of SCD to inform health-policy makers to implement preventive guidelines. Therefore, we investigated the association of anthropometric indices including body mass index (BMI), WC, WHR, WHtR, and HC with incident SCD among subjects with and without T2DM.

## Methods

### Study design and study population

This study was done within the framework of the Tehran Lipid and Glucose Study (TLGS). The TLGS is a prospective cohort study among a general population that resided in district 13 of Tehran. This study was originally designed to assess the epidemiologic aspects of non-communicable diseases (NCDs) and to prevent NCDs by advancing healthier lifestyles. The recruitment was conducted in two phases [January 31, 1999–July 03, 2001 (phase I) and October 20, 2001–September 22, 2005 (phase II)] and the TLGS was planned to continue for at least 20 years by follow-up phases with 3-year intervals. For the current study, we only used the data of enrollment phases (I and II) for our variables. Further details for the design, measurement methods, and enrollment strategy of the TLGS have been described elsewhere [20].

**Fig. 1:**
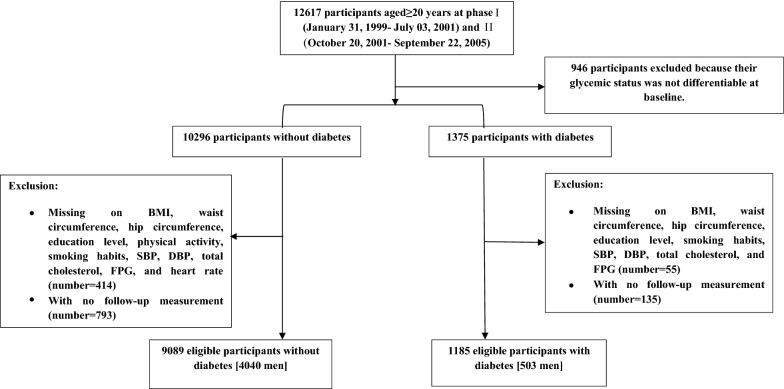
Flowchart of the study population. Tehran Lipid and Glucose Study, Iran, 1999–2018

As shown in Fig. 1, among a total of 12,617 individuals aged ≥ 20 years, initially, we excluded 946 participants whose diabetes status was not differentiable at baseline due to lack of data on fasting plasma glucose (FPG) or 2-h post-challenge plasma glucose (2 h-PCPG). Of the remaining 11,671 participants, 1375 participants were in T2DM group. Firstly, we excluded those with missing data on covariate (414 subjects without T2DM and 55 subjects with T2DM). Then we further excluded those with no follow-up measurement (793 subjects without T2DM and 135 subjects with T2DM). Finally, 9089 subjects without T2DM and 1185 subjects with T2DM remained eligible for our analysis.

### Clinical and laboratory measurements

Demographic data, past medical and drug history, family history of CVD, smoking habits, and education level were obtained by structured questionnaires at the enrollment phases. Subjects who were enrolled at phase I were asked to provide a subjective rating of their physical activity level using the Lipid Research Clinic (LRC) questionnaire [21]. The Modifiable Activity Questionnaire (MAQ) was used for subjects who were enrolled at phase II [20, 22] to assess physical activity level. The MAQ measures all three forms of activities including leisure time, job, and household activities in the past year. Weight was recorded with shoes removed and wearing light clothing to the nearest 100 g using digital scales (Seca 707, Seca Corp., Hanover, MD, USA; range 0.1–150 kg). While participants were asked to keep their shoulders in normal alignment, the height of the subjects was recorded in a standing position using a tape measure. BMI was calculated as weight divided by the square of the height (kg/m^2^). WC was measured at the umbilical level, using a tape meter, with no pressure on the body surface. HC was also measured at the maximal level over light clothing. WHR and WHtR were calculated as WC (cm) divided by HC (cm) and by height (cm), respectively. We defined the subject’s blood pressure (BP) as the mean of the two standardized measurements on the right arm by a sphygmomanometer after 15 min of rest. Pulse rate was recorded as a mean of two times counting pulse of the radial artery in one minute. Participants were asked to fast for at least 12 h before morning blood sample collection. Samples were analyzed on the same day in the TLGS laboratory. For measurement 2h-PCPG, 82.5 g glucose monohydrate solution (equivalent to 75 g anhydrous glucose) was orally taken by individuals that are not on glucose-lowering medications. Total cholesterol, 2h-PCPG, and FPG were measured by standard methods, as explained before [20].

### Definition of variables

T2DM was defined as the presence of at least one of the following criteria: (a) FPG level of ≥ 7.0 mmol/L (126 mg/dL), (b) 2 h-PCPG level of ≥ 11.1 mmol/L (200 mg/dL), (c) positive history of glucose-lowering medications usage [23]. Hypercholesterolemia was considered as having a total cholesterol level of ≥ 5.17 mmol/L (200 mg/dL) or use of lipid-lowering medications. In our study, hypertension was confirmed by having at least one of the following parameters: systolic blood pressure (SBP) ≥ 140 mmHg or diastolic blood pressure (DBP) ≥ 90 mmHg or positive history of antihypertensive medications usage. Smoking status was classified into never/former smoker versus current smokers. Individuals were categorized based on their education level: (a) participants who have less than 6 years of education; (b) ones with 6–12 years of education; (c) ones with more than 12 years of formal education. Being physically active in less than 3 days of each week for participants who were enrolled at phase I and having less than 600 METs (metabolic equivalent task-minutes) weekly for participants who were enrolled at phase II, considered as low physical activity [20, 24]. A positive family history of premature CVD for the participant was defined as prior diagnosed CVD in female first-degree relatives aged <65 years or male first-degree relatives aged <55 years.

### Outcome

Details of the data collection for outcome assessment have been published before [20, 25]. In brief, each individual was followed-up for any medical problem leading to hospitalization. As a part of the data gathering for the TLGS, a trained nurse called all individuals annually and recorded any medical events during the last year. By a home visit, a trained general practitioner followed-up any reported event and gathered complementary medical data from the hospital. Moreover, in the cases of death, information on the death certificate, the forensic medicine report, and the verbal autopsy was obtained. Then collected documents were evaluated by an outcome committee that included a principal investigator, an internist, an endocrinologist, a cardiologist, an epidemiologist, and if needed, other experts. After adjudication by the outcome committee, each event was assigned to a specific outcome. All of the fatal cases in TLGS were critically also evaluated by the outcome committee members. Definite SCD was defined as a sudden pulseless condition attributable to a cardiac origin in a previously stable individual. Possible SCD was known as unpredictable death, 24 h after last having observed alive that did not attributable to a specific source of circulatory collapse or an underlying source than heart disease. In this study, incident SCD was defined as cases of definite and possible SCD [15].

### Statistical analysis

Baseline characteristics of the study population were described as mean (standard deviation: SD) values for continuous variables and as frequencies (%) for categorical variables. The mean values and proportions of the baseline variables were compared between T2DM versus non-T2DM groups using Student’s two-tailed t-test and Chi-square tests, as appropriate; the similar approach was also applied to compare baseline characteristics of participants with SCD versus survivors in each of T2DM and non-T2DM groups, separately.

Hazard ratios (HRs) with 95% confidence intervals (CIs) were reported using Cox proportional hazard models to evaluate the association of different anthropometric indices with incident SCD. We considered anthropometric indices including BMI, WC, WHR, WHtR, and HC, as continuous and categorical variables in our models. Due to different measurement units among anthropometric indices, we determined the HRs according to a 1 SD increase in each anthropometric index, considering indices as continuous variables. To categorize the anthropometric indices, we calculated quartiles for each index in T2DM and non-T2DM group, separately, according to their distribution in the study population (considering the first quartile as reference group). Moreover, the P-value for trend was calculated by considering each quartile as a continuous variable.

Univariable Cox regression was performed among participants with and without T2DM separately for each potential risk factor including age, sex, smoking status, educational level, positive history of CVD, family history of premature CVD, hypertension, hypercholesterolemia, low physical activity, FPG level, and pulse rate. Those with a P-value of < 0.2 were selected to enter the multivariable models as covariates. HRs with 95% CIs are reported in two multivariable models for both T2DM and non-T2DM groups: Model 1 was adjusted for age and sex. Model 2 was further adjusted for current smoking, education level, family history of premature CVD, positive history of CVD, hypertension, hypercholesterolemia, and FPG level at baseline. In the non-T2DM group, low physical activity and pulse rate were also considered in model 2 as covariates.

We checked the interaction of each anthropometric index quartiles with T2DM status (yes or no) in the crude analysis [P-values were 0.141, 0.009, <0.001, <0.001, and 0.334 for BMI, WC, WHR, WHtR, and HC, respectively]. Hence we stratified our study population by the presence of T2DM.

The proportionality in the Cox model was evaluated with the Schoenfeld residual test. Generally, all proportionality assumptions were appropriate. The event date was defined as the date of the occurrence of SCD. Those who met the following criteria were considered to be censored: leaving the residential area, loss to follow-up, or end of follow-up. For individuals with SCD, survival time was defined as the time between the entered date and the death date. Additionally, for the censored participants, the survival time was defined as the difference between the entered date and the last available follow-up date (20 March 2018).

Statistical analyses were performed by STATA version 14 (StataCorp LP, College Station, Texas) statistical software. P-values < 0.05 were considered statistically significant.

## Results

Baseline characteristics of the participants with and without T2DM are presented in Table 1. The study population consisted of 9089 (4040 men) subjects without T2DM and 1185 (503 men) subjects with T2DM. The mean age (SD) of participants was 41.0 (13.9) years in the non-T2DM group and 55.5 (11.5) years in the T2DM group. Generally, compared to those without diabetes, subjects with T2DM were older and had higher values of SBP, DBP, pulse rate, FPG, and total cholesterol. Furthermore, all of the anthropometric indices were at a higher range among the T2DM group. Among categorical variables, the prevalence of current smoking and high educated level (> 12 years) were higher in non-T2DM participants. On the other hand, positive family history of premature CVD, having CVD, and using lipid-lowering/antihypertensive medications were more prevalent in participants with T2DM.

**Table 1:** Baseline characteristics of the participants with and without Type 2 diabetes mellitus: Tehran Lipid and Glucose Study, Iran, 1999–2018

Number of participants (men)	No diabetes	With diabetes	P-value*
	9089 (4040)	1185 (503)	
Continuous variables, Mean (SD)			
Age (year)	41.0 (13.9)	55.5 (11.5)	< 0.001
BMI (kg/m^2^)	26.6 (4.7)	28.9 (4.6)	< 0.001
WC (cm)	87.7 (12.0)	96.6 (10.9)	< 0.001
WHR	0.87 (0.09)	0.94 (0.08)	< 0.001
WHtR	0.54 (0.08)	0.61 (0.08)	< 0.001
HC (cm)	100.6 (9.4)	102.6 (9.8)	< 0.001
SBP (mmHg)	117.2 (17.7)	134.4 (22.9)	< 0.001
DBP (mmHg)	76.6 (10.8)	82.3 (11.7)	< 0.001
Pulse rate (beat/ minute)	79.0 (11.4)	80.0 (12.1)	0.004
FPG (mmol/L)⁑	5.0 (0.5)	9.1 (3.4)	< 0.001
Total cholesterol (mmol/L)⁑	5.2 (1.2)	6.0 (1.3)	< 0.001
Categorical variables, number (%)			
Current smoker	1507 (16.6)	154 (13.0)	0.002
Education level			< 0.001
Illiterate/primary school	2710 (29.8)		
Below diploma/diploma	5029 (55.3)	374 (31.6)	
Above diploma	1350 (14.9)	76 (6.4)	
Low physical activity, yes	6171 (67.9)	838 (70.7)	0.050
Family history of premature CVD, yes	1389 (15.3)	230 (19.4)	< 0.001
Prevalent CVD at baseline, yes	309 (3.4)	162 (13.7)	< 0.001
Lipid-lowering medication, yes	201 (2.2)	133 (11.2)	< 0.001
Antihypertensive medication, yes	504 (5.5)	273 (23)	< 0.001

*BMI* body mass index, *WC* waist circumference, *WHR* waist-to-hip ratio, *WHtR* waist-to-height ratio, *HC* hip circumference, *SBP* systolic blood pressure, *DBP* diastolic blood pressure, *FPG* fasting plasma glucose, *CVD* cardiovascular disease

Values are shown as Mean (standard deviation: SD) and number (%) for continuous and categorical variables, respectively

*The comparison P-value between two groups was calculated using Student’s two-tailed t-test for continues variables and Chi-square test for categorical variables

⁑Conversion factors from mmol/L to mg/dL were 18.02 for FPG and 38.67 for total cholesterol

Baseline characteristics of the participants stratified by outcome occurrence (SCD) during follow-up are presented in Table 2. Specifically, considering anthropometric indices, among participants without T2DM, those with incident SCD had higher values of WC, WHR, and WHtR, and a lower range of HC. Among participants with T2DM, those with SCD had higher WHR and lower HC; however, no difference was observed in the level of BMI, WC, and WHtR.

**Table 2:** Baseline characteristics of the participants, stratified by outcome occurrence (Sudden cardiac death: SCD) during follow-up: Tehran Lipid and Glucose Study, Iran, 1999–2018

	No Diabetes			With Diabetes		
	No-SCD	SCD	P-value*	No-SCD	SCD	P-value*
Number of participants (Men)	8945 (3930)	144 (110)		1099 (456)	86 (47)	
Continuous variables, Mean (SD)						
Age (year)	40.7 (13.7)	58.5 (14.0)	< 0.001	55.0 (11.5)	61.8 (8.7)	< 0.001
BMI (kg/m^2^)	26.6 (4.7)	26.9 (4.7)	0.575	28.9 (4.6)	28.2 (5.1)	0.167
WC (cm)	87.6 (12.0)	92.5 (11.5)	< 0.001	96.6 (10.8)	97.6 (11.6)	0.408
WHR	0.87 (0.09)	0.94 (0.07)	< 0.001	0.94 (0.08)	0.98 (0.08)	< 0.001
WHtR	0.54 (0.08)	0.57 (0.08)	< 0.001	0.61 (0.08)	0.61 (0.07)	0.733
HC (cm)	100.7 (9.4)	98.3 (9.4)	0.003	102.8 (9.8)	99.5 (9.6)	0.002
SBP (mmHg)	117.0 (17.4)	130.6 (25.1)	< 0.001	133.7 (22.5)	143.3 (25.9)	< 0.001
DBP (mmHg)	76.6 (10.7)	80.7 (14.2)	0.001	82.2 (11.5)	83.9 (13.4)	0.255
Pulse rate (beat/minute)	79.0 (11.4)	76.3 (13.4)	0.016	80.0 (12.0)	80.0 (12.6)	0.921
FPG (mmol/L)⁑	5.0 (0.5)	5.1 (0.5)	0.011	8.9 (3.3)	10.6 (3.8)	< 0.001
Total cholesterol (mmol/L)⁑	5.2 (1.2)	5.6 (1.2)	< 0.001	6.0 (1.3)	6.2 (1.2)	0.253
Categorical variables, number (%)						
Current smoker	1466 (16.4)	41 (28.5)	< 0.001	137 (12.5)	17 (19.8)	0.052
Education level			< 0.001			0.001
Illiterate/primary school	2624 (29.3)	86 (89.7)		666 (60.6)		
Below diploma/diploma	4979 (55.7)	50 (34.7)		364 (33.1)	11 (12.8)	
Above diploma	1342 (15)	8 (5.6)		69 (6.3)	7 (8.1)	
Low physical activity, yes	6062 (67.8)	109 (75.7)	0.043	772 (70.2)	66 (76.7)	0.202
Family history of premature CVD, yes	1359 (15.2)	30 (20.8)	0.062	222 (20.2)	8 (9.3)	0.014
Prevalent CVD at baseline, yes	287 (3.2)	22 (15.3)	< 0.001	142 (12.9)	20 (23.3)	0.007
Lipid-lowering medication, yes	194 (2.2)	7 (4.9)	0.029	125 (11.4)	8 (9.3)	0.558
Antihypertensive medication, yes	470 (5.3)	34 (23.6)	< 0.001	247 (22.5)	26 (30.2)	< 0.001

*BMI* body mass index, *WC* waist circumference, *WHR* waist-to-hip ratio, *WHtR* waist-to-height ratio, *HC* hip circumference, *SBP* systolic blood pressure, *DBP* diastolic blood pressure, *FPG* fasting plasma glucose, *CVD* cardiovascular disease

Values are shown as Mean (standard deviation: SD) and number (%) for continuous and categorical variables, respectively

*The comparison P-value between groups was calculated using Student’s two-tailed t-test for continues variables and Chi-square test for categorical variables

⁑Conversion factors from mmol/L to mg/dL were 18.02 for FPG and 38.67 for total cholesterol

### Non-T2DM group

**Fig. 2:**
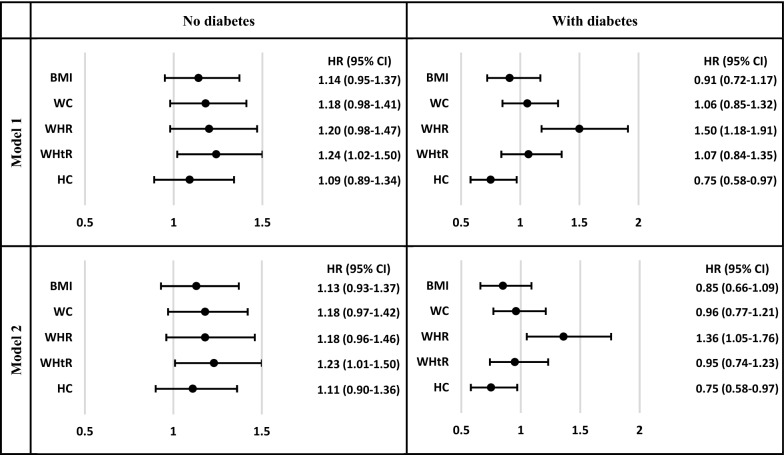
Multivariable hazard ratios (HR) and 95% confidence intervals (CI) of a 1 standard deviation (SD) increase in anthropometric indices (as continuous variables) for incident sudden cardiac death (SCD): Tehran Lipid and Glucose Study, Iran, 1999–2018

During a median follow-up of 17.9 years (interquartile range: IQR, 13.9–18.5), 144 incident SCD have occurred. A 1 SD increase in WHtR was associated with higher risk of incident SCD by significant HRs of 1.24 (95% CI 1.02–1.50) and 1.23 (1.00–1.50) in model 1 and 2, respectively (Fig. 2). Moreover, the trend of SCD risk from the first quartile to the fourth quartile of WHtR was significant in model 1 (P-value for trend: 0.041). The trend was also marginally significant in model 2 by a P-value of 0.055 (Table 3). Moreover, a 1 SD increase in WC and WHR tend to be associated with higher risk of incident SCD only in age- and sex-adjusted analysis [HR for WC: 1.18 (0.98–1.41), p-value: 0.084; HR for WC: 1.20 (0.98–1.47), P-value: 0.074] (Fig. 2). Other indices did not have any significant effect on incident SCD in the non-T2DM group.

**Table 3:** Multivariable hazard ratios (HR) and 95% confidence intervals (CI) of different anthropometric indices (as categorical variables) for incident sudden cardiac death (SCD) among participants without diabetes: Tehran Lipid and Glucose Study, Iran, 1999–2018

	Quartile range	E/N	Model 1		Model 2	
			HR (95% CI)	p-value	HR (95% CI)	p-value
BMI						
First quartile	< 23.4 Kg/m^2^	35/2283	Reference		Reference	
Second quartile	23.4–26.3 Kg/m^2^	34/2265	0.78 (0.48–1.24)	0.293	0.85 (0.53–1.38)	0.510
Third quartile	26.3–29.4 Kg/m^2^	39/2274	0.97 (0.62–1.54)	0.912	0.99 (0.62–1.60)	0.982
Fourth quartile	29.4 Kg/m^2^ ≤	36/2267	1.20 (0.74–1.95)	0.458	1.19 (0.71–2.00)	0.506
P-value for trend				0.338		0.426
WC						
First quartile	< 80 cm	18/2348	Reference		Reference	
Second quartile	80–88 cm	36/2443	1.04 (0.59–1.83)	0.902	1.11 (0.63–1.98)	0.713
Third quartile	89–97 cm	37/2170	1.01 (0.57–1.78)	0.966	1.08 (0.61–1.94)	0.787
Fourth quartile	97 cm ≤	53/2128	1.39 (0.81–.38)	0.233	1.43 (0.81–2.51)	0.214
P-value for trend				0.156		0.176
WHR						
First quartile	< 0.81	6/2281	Reference		Reference	
Second quartile	0.81–0.87	19/2277	1.43 (0.56–3.62)	0.453	1.36 (0.54–3.45)	0.513
Third quartile	0.870.93	43/2266	1.88 (0.77–4.55)	0.164	1.78 (0.74–4.32)	0.200
Fourth quartile	0.93 ≤	76/2265	1.90 (0.78–4.62)	0.155	1.75 (0.72–4.26)	0.215
P-value for trend				0.135		0.207
WHtR						
First quartile	< 0.48	18/2281	Reference		Reference	
Second quartile	0.48–0.54	31/2269	1.05 (0.59–1.88)	0.870	1.10 (0.61–1.99)	0.748
Third quartile	0.54–0.59	44/2268	1.23 (0.70–2.14)	0.471	1.31 (0.74–2.32)	0.354
Fourth quartile	0.59 ≤	51/2271	1.64 (0.93–2.87)	0.085	1.65 (0.92–2.98)	0.096
P-value for trend				**0.041**		0.055
HC						
First quartile	< 95 cm	51/2381	Reference		Reference	
Second quartile	95–101 cm	43/2519	0.98 (0.65–1.47)	0.905	1.01 (0.67–1.54)	0.951
Third quartile	101–107 cm	28/2128	1.13 (0.70–1.82)	0.618	1.14 (0.70–1.85)	0.609
Fourth quartile	107 cm ≤	22/2061	1.37 (0.77–2.42)	0.284	1.36 (0.75–2.47)	0.305
P-value for trend				0.311		0.327

*E* event, *N* number, *BMI* body mass index, *WC* waist circumference, *WHR* waist-to-hip ratio, *WHtR* waist-to-height ratio, *HC* hip circumference, *CVD* cardiovascular disease

Model 1 was adjusted for age and sex. Model 2 was further adjusted for current smoking, education level, positive history of cardiovascular disease, family history of premature cardiovascular disease, hypertension, hypercholesterolemia, low physical activity, FPG level, and pulse rate

### T2DM group

For the T2DM group, 86 incident SCD cases were also found during a median follow-up of 15.8 years (10.3–18.3). A 1 SD increase in WHR had HRs of 1.50 (1.18–1.91) and 1.36 (1.05–1.76) in model 1 and 2, respectively (Fig. 2). By considering WHR as categorical variables, the trend of SCD risk across quartiles of WHR was also significant in both models (Table 4). Furthermore, a 1 SD increase in HC led to reduced risk with a HR of 0.75 (0.58–0.97) in model 2 (Fig. 2). Compared to the first quartile, the fourth quartile of HC also showed a HR of 0.50 (0.25–0.99), and the trend of SCD risk was significant across quartiles (Table 4). As a sensitive analysis, after further adjustment for WC, the HR of 1 SD increase in HC changed to 0.54 (0.36–0.80), and second, third, and fourth quartiles of HC had HRs of 0.82 (0.46–1.46), 0.38 (0.18–0.83), and 0.27 (0.11–0.69), respectively, compared to the first quartile.

**Table 4:** Multivariable hazard ratios (HR) and 95% confidence intervals (CI) of different anthropometric indices (as categorical variables) for incident sudden cardiac death (SCD) among participants with diabetes: Tehran Lipid and Glucose Study, Iran, 1999–2018

	Quartile range	E/N	Model 1		Model 2	
			HR (95% CI)	p-value	HR (95% CI)	p-value
BMI						
First quartile	< 25.9 kg/m^2^	26/297	Reference		Reference	
Second quartile	25.9–28.5 kg/m^2^	25/296	0.89 (0.51–1.55)	0.684	0.86 (0.49–1.51)	0.610
Third quartile	28.5–31.6 kg/m^2^	17/297	0.67 (0.36–1.24)	0.205	0.69 (0.37–1.28)	0.235
Fourth quartile	31.6 kg/m^2^ ≤	18/295	0.83 (0.44–1.54)	0.546	0.68 (0.36–1.29)	0.237
P-value for trend				0.356		0.169
WC						
First quartile	< 91 cm	23/342	Reference		Reference	
Second quartile	91–98 cm	18/287	0.85 (0.46–1.58)	0.617	0.72 (0.39–1.35)	0.309
Third quartile	98–105 cm	26/279	1.15 (0.65–2.02)	0.628	1.12 (0.63–1.98)	0.395
Fourth quartile	105 cm ≤	19/277	0.89 (0.48–1.63)	0.700	0.72 (0.38–1.36)	0.316
P-value for trend				0.970		0.640
WHR						
First quartile	< 0.90	11/299	Reference		Reference	
Second quartile	0.90–0.95	12/298	0.87 (0.38–1.98)	0.735	0.79 (0.35–1.81)	0.583
Third quartile	0.95–1.01	31/333	1.82 (0.89–3.70)	0.099	1.53 (0.75–3.13)	0.240
Fourth quartile	1.01 ≤	32/255	**2.27 (1.10–4.69)**	**0.027**	1.76 (0.84–3.70)	0.137
P-value for trend				**0.003**		**0.033**
WHtR						
First quartile	< 0.55	18/297	Reference		Reference	
Second quartile	0.55–0.60	28/297	1.38 (0.76–2.51)	0.288	1.28 (0.70–2.33)	0.423
Third quartile	0.60–0.65	19/297	1.01 (0.52–1.95)	0.976	0.93 (0.48–1.81)	0.827
Fourth quartile	0.65 ≤	21/294	1.23 (0.62–2.47)	0.552	0.97 (0.48–1.95)	0.937
P-value for trend				0.821		0.683
HC						
First quartile	< 97 cm	35/343	Reference		Reference	
Second quartile	97–103 cm	26/302	0.93 (0.56–1.55)	0.776	1.05 (0.62–1.77)	0.848
Third quartile	103–109 cm	13/255	0.53 (0.28–1.02)	0.059	0.57 (0.29–1.09)	0.090
Fourth quartile	109 cm ≤	12/285	0.51 (0.26–1.03)	0.059	**0.50 (0.25–0.99)**	**0.047**
P-value for trend				**0.020**		**0.018**

*E* event, *N* number, *BMI* body mass index, *WC* waist circumference, *WHR* waist-to-hip ratio, *WHtR* waist-to-height ratio, *HC* hip circumference, *CVD* cardiovascular disease

Model 1 was adjusted for age and sex. Model 2 was further adjusted for current smoking, education level, positive history of cardiovascular disease, family history of premature cardiovascular disease, hypertension, hypercholesterolemia, and FPG level

Importantly, whether considering anthropometric indices as continuous or categorical variables, being male, older age, family history of CVD, having history of CVD at baseline, and current smoking were significantly associated with incident SCD in all full-adjusted models 2 (data not shown).

After sex stratification in our sensitivity analysis, no significant association was found between anthropometric indices, whether as continuous or categorical variables, with incident SCD in both T2DM and non-T2DM groups, except that a 1 SD increase in WHR was associated with higher risk of incident SCD among diabetic men (Additional file 1: Fig.S1, Additional file 2: Fig.S2 Additional file 3: Table S1, Additional file 4: Table S2, Additional file 5: Table S3 and Additional file 6: Table S4)

## Discussion

The current study is a prospective population-based study that examined the association of five anthropometric indices with incident SCD in both T2DM and non-T2DM groups over 15 years of follow-up. In the multivariable analysis, adjusted for well-known SCD risk factors, in subjects without T2DM, higher WHtR had a positive association with incident SCD; among subjects with T2DM, on the other hand, increasing in the WHR and HC value was associated with increased and decreased risk, respectively.

We did not find any significant association between general obesity and incident SCD among both T2DM and non-T2DM groups, as similarly shown in some studies [26, 27]. Nevertheless, a recent meta-analysis pointed out that a 5-unit increment in BMI was associated with a 16% higher risk of SCD; however, there is a high heterogeneity between included studies [12]. Other meta-analysis found that compared to normal weight status, obesity was associated with incident SCD by a relative risk of 1.52 (1.31–1.77) [13]. In our data analysis, among the non-T2DM group, compared to those with BMI less than 23.4 kg/m^2^, participants with BMI ≥ 29.4 kg/m^2^ had about a 20% non-significant higher risk.

We demonstrated that WC did not significantly increase the risk of incident SCD. Likely, in a study among male residents in Eastern Finland, a non-significant association between WC and incident SCD was observed [28]. In the study of Adabag et al., among a non-smoker population, a significant trend was found between increasing values of WC and SCD in the confounder adjusted model; however, after further adjustment for SCD mediators, this association was disappeared [15].

In the current study, we found that increasing value of WHR was significantly associated with incident SCD in T2DM group; the risk was more prominent among male participants. Among post-menopausal women, Bertoia et al. found WHR to be associated positively with SCD risk; they reported that compared to the first quartile, the risk of those in the fourth quartile of WHR was increased by about 70% [29]. In a recent meta-analysis, using data from three cohort studies, it was highlighted that a 0.1-unit increment in WHR was associated with a 82% increased risk of incident SCD (95% CI 1.61–2.07), with zero heterogeneity between included studies. Moreover, the researchers demonstrated a non-linear association between increasing values of WHR and incident SCD; however, they didn’t show any threshold effect [12].

We found out that non-diabetic individuals with higher WHtR values were significantly more susceptible to SCD. Up to now, this association was not investigated by any other study. By including more than 3,00,000 participants in the meta-analysis, Ashwel et al. showed that WHtR provided preferred tools for discriminating different cardiometabolic risks, including CVD, compared to BMI and WC [30].

The responsible mechanism for increasing the risk of CVD in individuals with central obesity has been studied. Central obesity has considerable effects on inflammation, which is postulated to be more prominent than general obesity [31]. It has been reported in support of this idea that inflammation may play a pivotal role in patients with [32, 33] and without [34, 35] macroscopic cause of SCD. Ayman et al. claimed that interleukin 6 (IL-6), a well-known marker of inflammation, has a significant positive association with SCD risk even following adjustment for two main SCD risk factors, including incident myocardial infarction and heart failure [36]. Importantly, a positive correlation was shown between IR and most adipose tissue depots/obesity indices, with the strongest association for visceral fat mass [37]. Moreover, central obesity was significantly associated with IR, contributing to cardiomyopathy and incident SCD through several pathways. First, due to limited glucose uptake, more fatty acid was metabolized by myocytes as a source of energy; it can decrease contractility by functional alterations at the mitochondrial level. Second, high circulating insulin levels stimulates insulin-like growth factor 1 (IGF-1) receptor; it can induce left ventricle hypertrophy [9].

In our study, higher HC had an association with lower risk of incident SCD in the T2DM group but not non-T2DM. In the atherosclerosis risk in communities (ARIC) study, Adabag et al. did not reach any significant association between HC and incident SCD in the US general population [15]. There are heterogeneous findings regarding the association between HC and CVD. According to the prospective studies, Heitmann et al. concluded that small hip size after controlling for general and/or central adiposity was associated with adverse cardiovascular outcomes [38]. Cameron et al. declared that without considering WC in risk models, HC acts as a detrimental factor; it turns into a protective one if WC included in CVD and mortality risk models [39]. In the current study, among subjects with T2DM, HC had a protective effect on incident SCD, even after including WC in the risk model. Similarly, in a study on South Asian and African Mauritians, Cameron et al. found a similar protective effect for higher HC on CVD mortality prior and after adjustment for WC; however, this finding was found only among women. Several mechanisms have been suggested for the protective role of high values HC on CVD events. Following reports of experimental studies, secretion of adipokines, especially leptin and adiponectin, from the fat tissues of gluteofemoral may be the mechanism behind the protective effects of HC on incident CVD [40]. Another possible mechanism is attributed to lipoprotein lipase activity, which is greater in fat tissues of the femoral region than visceral [41].

The major strength of our study is that we assessed the association of five main anthropometric indices with the risk of incident SCD in both T2DM and non-T2DM groups by a prospective design, large scale study, and a long follow-up period in the MENA region. Another strength is that we adjusted the associations for the precise measured, but not self-reported SCD risk factors.

Several limitations need to be acknowledged. First, we didn’t have data on prior history of non-ischemic heart diseases such as structural heart disease or arrhythmic syndrome, the important potential risk factors and mediators of incident SCD [7]; hence we did not consider them in our data analysis. Second, we included the baseline data only and did not assess the association of trajectory or change of anthropometric indices. Third, there is a chance that death coded as SCD may not be due to that, and may because of other reasons, such as cerebral hemorrhage and pulmonary embolisms. Fourth, we have no data on inflammatory markers of visceral adiposity, including highly sensitive CRP, IL-6, and WBC in this population-based study. Fifth, at recruitment of the TLGS, food and drink consumption were not assessed for study population and last but not least, our study population is limited to residents of Tehran, a metropolitan city, with uniform ethnicity; hence our findings may not be generalizable to rural populations or other ethnicities. Further studies are needed to examine the association of anthropometric indices and incident SCD in other populations.

In conclusion, first, we demonstrated central but not general obesity (as assessed by WHR in participants with T2DM and WHtR in participants without T2DM) as a herald of incident SCD. Second, HC, whether adjusted for WC or not, can have an inverse association with incident SCD among participants with T2DM. It can be realized that central obesity can associated more with incident SCD than general obesity. Third, WHR and WHtR better capture those with higher risk of SCD than WC. Third, the effect of anthropometric indices on incident SCD can be differed among diabetic and non-diabetic participants. Fourth, for decreasing the risk of incident SCD, interventions for the obesity management (especially central obesity), through life style changes and pharmacological or surgical approaches, should be taken into account. Beside to losing weight, cardiorespiratory fitness (CRF) is more closely related to CVD outcomes and a better predictor of cardiac outcomes [9]. Therefore, studies with aim of assessing the impact of CRF changes give us a better view. Finally, in the current study, we did not assess the influence of dynamic changes of obesity on SCD incident; hence it is necessary to conduct studies that evaluate the impact of dynamic changes of obesity indices on incident SCD.

## Supplementary Information

**Additional file 1:** **Figure S1.** Multivariable hazard ratios (HR) and 95% confidence intervals (CI) of a 1 standard deviation (SD)* increase in anthropometric indices (as continuous variables) for incident sudden cardiac death (SCD) among female participants: Tehran Lipid and Glucose Study, Iran, 1999-2018.

**Additional file 2:** **Figure S2.** Multivariable hazard ratios (HR) and 95% confidence intervals (CI) of a 1 standard deviation (SD) increase in anthropometric indices (as continuous variables) for incident sudden cardiac death (SCD) among male participants: Tehran Lipid and Glucose Study, Iran, 1999-2018.

**Additional file 3:** **Table S1.** Multivariable hazard ratios (HR) and 95% confidence intervals (CI) of different anthropometric indices (as categorical variables) for incident sudden cardiac death (SCD) among female participants without diabetes: Tehran Lipid and Glucose Study, Iran, 1999-2018.

**Additional file 4:** **Table S2.** Multivariable hazard ratios (HR) and 95% confidence intervals (CI) of different anthropometric indices (as categorical variables) for incident sudden cardiac death (SCD) among male participants without diabetes: Tehran Lipid and Glucose Study, Iran, 1999-2018.

**Additional file 5:** **Table S3.** Multivariable hazard ratios (HR) and 95% confidence intervals (CI) of different anthropometric indices (as categorical variables) for incident sudden cardiac death (SCD) among female participants with diabetes: Tehran Lipid and Glucose Study, Iran, 1999-2018.

**Additional file 6:** **Table S4.** Multivariable hazard ratios (HR) and 95% confidence intervals (CI) of different anthropometric indices (as categorical variables) for incident sudden cardiac death (SCD) among male participants with diabetes: Tehran Lipid and Glucose Study, Iran, 1999-2018.

## Data Availability

The datasets used and analyzed during the current study are available from the corresponding author on reasonable request.

## Abbreviations

CVD: Cardiovascular disease
SCD: Sudden cardiac death
CHD: Coronary heart disease
DM: Diabetes mellitus
IR: Insulin resistance
WC: Waist circumference
WHR: Waist-to-hip circumference
WHtR: Waist-to-height ratio
HC: Hip circumference
T2DM: Type 2 diabetes mellitus
BMI: Body mass index
TLGS: Tehran Lipid and Glucose Study
NCD: Non-communicable disease
FPG: Fasting plasma glucose
2 h-PCPG: 2-H post challenge plasma glucose
LRC: Lipid research clinic
MAQ: Modifiable activity questionnaire
BP: Blood pressure
SBP: Systolic blood pressure
DBP: Diastolic blood pressure
METs: Metabolic equivalent task-minutes
SD: Standard deviation
HR: Hazard ratio
CI: Confidence interval
IQR: Interquartile range
IL-6: Interleukin 6
IGF-1: Insulin-like growth factor 1
ARIC: Atherosclerosis risk in communities
CRF: Cardiorespiratory fitness

## References

[1] Roth GA, Mensah GA, Johnson CO, Addolorato G, Ammirati E, Baddour LM (2020). Global burden of cardiovascular diseases and risk factors, 1990–2019: update from the GBD 2019 study. J Am Coll Cardiol.

[2] Sarrafzadegan N, Mohammmadifard N (2019). Cardiovascular disease in Iran in the last 40 years: prevalence, mortality, morbidity, challenges and strategies for cardiovascular prevention. Arch Iran Med.

[3] Mehra R (2007). Global public health problem of sudden cardiac death. J Electrocardiol.

[4] Zheng Z-J, Croft JB, Giles WH, Mensah GA (2001). Sudden cardiac death in the United States, 1989 to 1998. Circulation.

[5] Adabag AS, Luepker RV, Roger VL, Gersh BJ (2010). Sudden cardiac death: epidemiology and risk factors. Nat Rev Cardiol.

[6] Moazzeni SS, Ghafelehbashi H, Hasheminia M, Parizadeh D, Ghanbarian A, Azizi F (2020). Sex-specific prevalence of coronary heart disease among Tehranian adult population across different glycemic status: Tehran lipid and glucose study, 2008–2011. BMC Public Health.

[7] Wong CX, Brown A, Lau DH, Chugh SS, Albert CM, Kalman JM (2019). Epidemiology of sudden cardiac death: global and regional perspectives. Heart Lung Circ.

[8] Myerburg RJ (2002). Scientific gaps in the prediction and prevention of sudden cardiac death. J Cardiovasc Electrophysiol.

[9] Plourde B, Sarrazin J-F, Nault I, Poirier P (2014). Sudden cardiac death and obesity. Expert Rev Cardiovasc Ther.

[10] Ormazabal V, Nair S, Elfeky O, Aguayo C, Salomon C, Zuñiga FA (2018). Association between insulin resistance and the development of cardiovascular disease. Cardiovasc Diabetol.

[11] Kim SH, Abbasi F (2019). Myths about insulin resistance: tribute to Gerald Reaven. Endocrinol Metab.

[12] Aune D, Schlesinger S, Norat T, Riboli E (2018). Body mass index, abdominal fatness, and the risk of sudden cardiac death: a systematic review and dose-response meta-analysis of prospective studies. Eur J Epidemiol.

[13] Chen H, Deng Y, Li S (2019). Relation of body mass index categories with risk of sudden cardiac death. Int Heart J.

[14] Agbaedeng T, Mahajan R, Munawar D, Elliott A, Twomey D, Khokhar K (2017). Obesity associates with increased risk of sudden cardiac death: a systematic review and meta-analysis. Heart Lung Circ.

[15] Adabag S, Huxley RR, Lopez FL, Chen LY, Sotoodehnia N, Siscovick D (2015). Obesity related risk of sudden cardiac death in the atherosclerosis risk in communities study. Heart.

[16] Kocovski L, Lee JD, Parpia S, Fernandes J, Nair V (2017). Association of waist-hip ratio to sudden cardiac death and severe coronary atherosclerosis in medicolegal autopsies. Am J Forensic Med Pathol.

[17] Aune D, Schlesinger S, Norat T, Riboli E (2018). Diabetes mellitus and the risk of sudden cardiac death: a systematic review and meta-analysis of prospective studies. Nutr Metab Cardiovasc Dis.

[18] Abarca-Gómez L, Abdeen ZA, Hamid ZA, Abu-Rmeileh NM, Acosta-Cazares B, Acuin C (2017). Worldwide trends in body-mass index, underweight, overweight, and obesity from 1975 to 2016: a pooled analysis of 2416 population-based measurement studies in 128 9 million children, adolescents, and adults. Lancet.

[19] Ghoorah K, Campbell P, Kent A, Maznyczka A, Kunadian V (2016). Obesity and cardiovascular outcomes: a review. Eur Heart J Acute Cardiovasc Care.

[20] Azizi F, Ghanbarian A, Momenan AA, Hadaegh F, Mirmiran P, Hedayati M (2009). Prevention of non-communicable disease in a population in nutrition transition: tehran lipid and glucose study phase II. Trials.

[21] Ainsworth BE, Jacobs DR, Leon AS (1993). Validity and reliability of self-reported physical activity status: the lipid research clinics questionnaire. Med Sci Sports Exerc.

[22] Momenan AA, Delshad M, Sarbazi N, Rezaei Ghaleh N, Ghanbarian A, Azizi F (2012). Reliability and validity of the modifiable activity questionnaire (MAQ) in an Iranian urban adult population. Arch Iran Med.

[23] Organization WH. Definition and diagnosis of diabetes mellitus and intermediate hyperglycaemia: report of a WHO/IDF consultation. 2006.

[24] The IPAQ Group. Guidelines for data processing and analysis of the International Physical Activity Questionnaire (IPAQ)-short and long forms. http://www.ipaq.ki.se. Accessed 27 Jul 2021.

[25] Khalili D, Azizi F, Asgari S, Zadeh-Vakili A, Momenan AA, Ghanbarian A (2018). Outcomes of a longitudinal population-based cohort study and pragmatic community trial: findings from 20 years of the Tehran Lipid and Glucose Study. Int J Endocrinol Metab.

[26] Ohira T, Maruyama M, Imano H, Kitamura A, Kiyama M, Okada T (2012). Risk factors for sudden cardiac death among Japanese: the Circulatory Risk in Communities Study. J Hypertens.

[27] Kataoka M, Ito C, Sasaki H, Yamane K, Kohno N (2004). Low heart rate variability is a risk factor for sudden cardiac death in type 2 diabetes. Diabetes Res Clin Pract.

[28] Karppi J, Laukkanen JA, Mäkikallio TH, Ronkainen K, Kurl S (2013). Serum β-carotene and the risk of sudden cardiac death in men: a population-based follow-up study. Atherosclerosis.

[29] Bertoia ML, Allison MA, Manson JE, Freiberg MS, Kuller LH, Solomon AJ (2012). Risk factors for sudden cardiac death in post-menopausal women. J Am Coll Cardiol.

[30] Ashwell M, Gunn P, Gibson S (2012). Waist-to-height ratio is a better screening tool than waist circumference and BMI for adult cardiometabolic risk factors: systematic review and meta-analysis. Obes Rev.

[31] Panagiotakos DB, Pitsavos C, Yannakoulia M, Chrysohoou C, Stefanadis C (2005). The implication of obesity and central fat on markers of chronic inflammation: the ATTICA study. Atherosclerosis.

[32] Manfredini R, Portaluppi F, Grandi E, Fersini C, Gallerani M (1996). Out-of-hospital sudden death referring to an emergency department. J Clin Epidemiol.

[33] Sudha ML, Sundaram S, Purushothaman KR, Kumar PS, Prathiba D (2009). Coronary atherosclerosis in sudden cardiac death: an autopsy study. Indian J Pathol Microbiol.

[34] Lecomte D, Fornes P, Fouret P, Nicolas G (1993). Isolated myocardial fibrosis as a cause of sudden cardiac death and its possible relation to myocarditis. J Forensic Sci.

[35] Wang HY, Huang WL, Yang CF, Song LF, Zhao H, Ren JM (2007). Morphologic features of sudden cardiac death in Yunnan province, with emphasis on myocarditis. Zhonghua Bing Li Xue Za Zhi.

[36] Hussein AA, Gottdiener JS, Bartz TM, Sotoodehnia N, DeFilippi C, See V (2013). Inflammation and sudden cardiac death in a community-based population of older adults: the Cardiovascular Health Study. Heart Rhythm.

[37] Zhang M, Hu T, Zhang S, Zhou L (2015). Associations of different adipose tissue depots with insulin resistance: a systematic review and meta-analysis of observational studies. Sci Rep.

[38] Heitmann BL, Lissner L (2011). Hip Hip Hurrah! Hip size inversely related to heart disease and total mortality. Obes Rev.

[39] Cameron A, Magliano D, Söderberg S (2013). A systematic review of the impact of including both waist and hip circumference in risk models for cardiovascular diseases, diabetes and mortality. Obes Rev.

[40] Manolopoulos KN, Karpe F, Frayn KN (2010). Gluteofemoral body fat as a determinant of metabolic health. Int J Obes.

[41] Rebuffé-Scrive M, Enk L, Crona N, Lönnroth P, Abrahamsson L, Smith U (1985). Fat cell metabolism in different regions in women. Effect of menstrual cycle, pregnancy, and lactation. J Clin Invest.

