# Supplementary material for: Anthropometric indices and the risk of incident sudden cardiac death among adults with and without diabetes: over 15 years of follow-up in The Tehran Lipid and Glucose Study
Source: Diabetol Metab Syndr. 2021 Jul 28;13:82. doi: 10.1186/s13098-021-00701-z (PMC8320203; doi:10.1186/s13098-021-00701-z)
Supplement: Supplementary file 2 — Additional file 2: Figure S2. Multivariable hazard ratios (HR) and 95% confidence intervals (CI) of a 1 standard deviation (SD) increase in anthropometric indices (as continuous variables) for incident sudden cardiac death (SCD) among male participants: Tehran Lipid and Glucose Study, Iran, 1999-2018. [file 13098_2021_701_MOESM2_ESM.docx]

| **Supplementary Figure 2. Multivariable hazard ratios (HR) and 95% confidence intervals (CI) of a 1 standard deviation (SD) increase in anthropometric indices (as continuous variables) for incident sudden cardiac death (SCD) among male participants: Tehran Lipid and Glucose Study, Iran, 1999-2018.** | | |
| --- | --- | --- |
|  | **No diabetes** | **With diabetes** |
| **Model 1** |  |  |
| **Model 2** |  |  |
| BMI: body mass index; WC: waist circumference; WHR: waist-to-hip ratio; WHtR: waist-to-height ratio; HC: hip circumference.  Model 1 was adjusted for age and sex. Model 2 was further adjusted for current smoking, education level, positive history of cardiovascular disease, family history of premature cardiovascular disease, hypertension, hypercholesterolemia, and FPG level. Among participants without diabetes, low physical activity and pulse rate were also considered in model 2.  *SDs were 4.09 and 3.98 kg/m^2^ for BMI, 11.16 and 10.48 cm for waist, 0.07 and 0.07 for WHR, 0.07 and 0.06 for WHtR, and 7.15 and 7.10 cm for HC among non-diabetic and diabetic participants, respectively. | | |
