# Supplementary material for: Anthropometric indices and the risk of incident sudden cardiac death among adults with and without diabetes: over 15 years of follow-up in The Tehran Lipid and Glucose Study
Source: Diabetol Metab Syndr. 2021 Jul 28;13:82. doi: 10.1186/s13098-021-00701-z (PMC8320203; doi:10.1186/s13098-021-00701-z)
Supplement: Supplementary file 3 — Additional file 3: Table S1. Multivariable hazard ratios (HR) and 95% confidence intervals (CI) of different anthropometric indices (as categorical variables) for incident sudden cardiac death (SCD) among female participants without diabetes: Tehran Lipid and Glucose Study, Iran, 1999-2018. [file 13098_2021_701_MOESM3_ESM.docx]

| **Supplementary Table 1. Multivariable hazard ratios (HR) and 95% confidence intervals (CI) of different anthropometric indices (as categorical variables) for incident sudden cardiac death (SCD) among female participants without diabetes: Tehran Lipid and Glucose Study, Iran, 1999-2018.** | | | | | | | |
| --- | --- | --- | --- | --- | --- | --- | --- |
|  | **Quartile Range** | | **E/N** | **Model 1** | | **Model 2** | |
|  |  |  |  | **HR (95% CI)** | **p-value** | **HR (95% CI)** | **p-value** |
| **BMI** | | | |  |  |  |  |
| **First Quartile** | < 23.4 Kg/m^2^ | 5/1101 | | **Reference** |  | **Reference** |  |
| **Second Quartile** | 23.4-26.3 Kg/m^2^ | 5/1114 | | 0.56 (0.16-1.93) | 0.356 | 0.53 (0.15-1.88) | 0.328 |
| **Third Quartile** | 26.3-29.4 Kg/m^2^ | 7/1232 | | 0.64 (0.20-2.02) | 0.443 | 0.53 (0.16-1.73) | 0.291 |
| **Fourth Quartile** | 29.4 Kg/m^2^ ≤ | 17/1602 | | 1.06 (0.39-2.90) | 0.906 | 0.82 (0.28-2.42) | 0.725 |
| **P-value for trend** |  |  | |  | 0.468 |  | 0.844 |
| **WC** | | | |  |  |  |  |
| **First Quartile** | < 80 cm | 4/1487 | | **Reference** |  | **Reference** |  |
| **Second Quartile** | 80-88 cm | 6/1343 | | 0.88 (0.25-3.12) | 0.842 | 0.85 (0.24-3.05) | 0.803 |
| **Third Quartile** | 89-97 cm | 9/1066 | | 1.20 (0.37-3.90) | 0.760 | 1.13 (0.34-3.75) | 0.836 |
| **Fourth Quartile** | 97 cm ≤ | 15/1153 | | 1.16 (0.38-3.51) | 0.794 | 0.92 (0.29-2.88) | 0.885 |
| **P-value for trend** |  |  | |  | 0.625 |  | 0.980 |
| **WHR** | | | |  |  |  |  |
| **First Quartile** | < 0.81 | | 4/2025 | **Reference** |  | **Reference** |  |
| **Second Quartile** | 0.81-0.87 | | 7/1462 | 1.04 (0.30-3.58) | 0.950 | 1.08 (0.31-3.73) | 0.901 |
| **Third Quartile** | 0.87-0.93 | | 10/975 | 1.20 (0.37-3.93) | 0.758 | 1.18 (0.36-3.86) | 0.781 |
| **Fourth Quartile** | 0.93 ≤ | | 13/587 | 1.45 (0.45-4.67) | 0.535 | 1.29 (0.39-4.21) | 0.675 |
| **P-value for trend** |  | |  |  | 0.438 |  | 0.634 |
| **WHtR** | | | |  |  |  |  |
| **First Quartile** | < 0.48 | | 1/1114 | **Reference** |  | **Reference** |  |
| **Second Quartile** | 0.48-0.54 | | 1/1099 | 0.43 (0.03-6.84) | 0.547 | 0.40 (0.02-6.39) | 0.513 |
| **Third Quartile** | 0.54-0.59 | | 9/1143 | 2.26 (0.28-18.0) | 0.440 | 2.18 (0.27-17.6) | 0.464 |
| **Fourth Quartile** | 0.59 ≤ | | 23/1693 | 2.09 (0.28-15.8) | 0.475 | 1.74 (0.22-13.5) | 0.598 |
| **P-value for trend** |  | |  |  | **0.156** |  | 0.269 |
| **HC** | | | |  |  |  |  |
| **First Quartile** | < 95 cm | | 6/772 | **Reference** |  | **Reference** |  |
| **Second Quartile** | 95-101 cm | | 5/1161 | 0.55 (0.17-1.80) | 0.322 | 0.49 (0.15-1.64) | 0.248 |
| **Third Quartile** | 101-107 cm | | 8/1340 | 0.77 (0.27-2.25) | 0.639 | 0.65 (0.22-1.93) | 0.440 |
| **Fourth Quartile** | 107 cm ≤ | | 15/1776 | 1.12 (0.43-2.96) | 0.813 | 0.89 (0.32-2.47) | 0.830 |
| **P-value for trend** |  | |  |  | 0.447 |  | 0.746 |
| E: event; N: number; BMI: body mass index; WC: waist circumference; WHR: waist-to-hip ratio; WHtR: waist-to-height ratio; HC: hip circumference; CVD: cardiovascular disease.  Model 1 was adjusted for age. Model 2 was further adjusted for current smoking, education level, positive history of cardiovascular disease, family history of premature cardiovascular disease, hypertension, hypercholesterolemia, low physical activity, FPG level, and pulse rate. | | | | | | | |
