# Supplementary material for: Anthropometric indices and the risk of incident sudden cardiac death among adults with and without diabetes: over 15 years of follow-up in The Tehran Lipid and Glucose Study
Source: Diabetol Metab Syndr. 2021 Jul 28;13:82. doi: 10.1186/s13098-021-00701-z (PMC8320203; doi:10.1186/s13098-021-00701-z)
Supplement: Supplementary file 4 — Additional file 4: Table S2. Multivariable hazard ratios (HR) and 95% confidence intervals (CI) of different anthropometric indices (as categorical variables) for incident sudden cardiac death (SCD) among male participants without diabetes: Tehran Lipid and Glucose Study, Iran, 1999-2018. [file 13098_2021_701_MOESM4_ESM.docx]

| **Supplementary Table 2. Multivariable hazard ratios (HR) and 95% confidence intervals (CI) of different anthropometric indices (as categorical variables) for incident sudden cardiac death (SCD) among male participants without diabetes: Tehran Lipid and Glucose Study, Iran, 1999-2018.** | | | | | | | |
| --- | --- | --- | --- | --- | --- | --- | --- |
|  | **Quartile Range** | | **E/N** | **Model 1** | | **Model 2** | |
|  |  |  |  | **HR (95% CI)** | **p-value** | **HR (95% CI)** | **p-value** |
| **BMI** | | | |  |  |  |  |
| **First Quartile** | < 23.4 Kg/m^2^ | 30/1182 | | **Reference** |  | **Reference** |  |
| **Second Quartile** | 23.4-26.3 Kg/m^2^ | 29/1151 | | 0.81 (0.49-1.36) | 0.429 | 0.95 (0.56-1.60) | 0.839 |
| **Third Quartile** | 26.3-29.4 Kg/m^2^ | 32/1042 | | 1.04 (0.63-1.72) | 0.870 | 1.17 (0.70-1.97) | 0.543 |
| **Fourth Quartile** | 29.4 Kg/m^2^ ≤ | 19/665 | | 1.12 (0.63-1.99) | 0.700 | 1.25 (0.68-2.30) | 0.481 |
| **P-value for trend** |  |  | |  | 0.555 |  | 0.366 |
| **WC** | | | |  |  |  |  |
| **First Quartile** | < 80 cm | 14/861 | | **Reference** |  | **Reference** |  |
| **Second Quartile** | 80-88 cm | 30/1100 | | 1.10 (0.58-2.08) | 0.777 | 1.27 (0.67-2.44) | 0.464 |
| **Third Quartile** | 89-97 cm | 28/1104 | | 0.97 (0.51-1.84) | 0.918 | 1.15 (0.59-2.24) | 0.676 |
| **Fourth Quartile** | 97 cm ≤ | 38/975 | | 1.41 (0.76-2.61) | 0.275 | 1.66 (0.87-3.17) | 0.126 |
| **P-value for trend** |  |  | |  | 0.261 |  | 0.139 |
| **WHR** | | | |  |  |  |  |
| **First Quartile** | < 0.81 | | 2/256 | **Reference** |  | **Reference** |  |
| **Second Quartile** | 0.81-0.88 | | 12/815 | 1.34 (0.30-5.98) | 0.706 | 1.41 (0.31-6.36) | 0.651 |
| **Third Quartile** | 0.88-0.93 | | 33/1291 | 1.69 (0.40-7.08) | 0.473 | 1.89 (0.45-7.99) | 0.385 |
| **Fourth Quartile** | 0.93 ≤ | | 63/1678 | 1.65 (0.40-6.82) | 0.491 | 1.82 (0.44-7.59) | 0.412 |
| **P-value for trend** |  | |  |  | 0.452 |  | 0.384 |
| **WHtR** | | | |  |  |  |  |
| **First Quartile** | < 0.48 | | 17/1167 | **Reference** |  | **Reference** |  |
| **Second Quartile** | 0.48-0.54 | | 30/1170 | 1.14 (0.63-2.07) | 0.670 | 1.24 (0.67-2.27) | 0.494 |
| **Third Quartile** | 0.54-0.59 | | 35/1125 | 1.15 (0.64-2.07) | 0.638 | 1.32 (0.72-2.42) | 0.378 |
| **Fourth Quartile** | 0.59 ≤ | | 28/578 | 1.56 (0.85-2.89) | 0.152 | 1.74 (0.91-3.32) | 0.092 |
| **P-value for trend** |  | |  |  | **0.163** |  | 0.094 |
| **HC** | | | |  |  |  |  |
| **First Quartile** | < 95 cm | | 45/1609 | **Reference** |  | **Reference** |  |
| **Second Quartile** | 95-101 cm | | 38/1358 | 1.03 (0.67-1.60) | 0.878 | 1.12 (0.72-1.75) | 0.610 |
| **Third Quartile** | 101-107 cm | | 20/788 | 1.16 (0.68-1.97) | 0.586 | 1.26 (0.73-2.17) | 0.417 |
| **Fourth Quartile** | 107 cm ≤ | | 7/285 | 1.32 (0.59-2.96) | 0.492 | 1.48 (0.65-3.37) | 0.354 |
| **P-value for trend** |  | |  |  | 0.446 |  | 0.275 |
| E: event; N: number; BMI: body mass index; WC: waist circumference; WHR: waist-to-hip ratio; WHtR: waist-to-height ratio; HC: hip circumference; CVD: cardiovascular disease.  Model 1 was adjusted for age. Model 2 was further adjusted for current smoking, education level, positive history of cardiovascular disease, family history of premature cardiovascular disease, hypertension, hypercholesterolemia, low physical activity, FPG level, and pulse rate. | | | | | | | |
