# Supplementary material for: Anthropometric indices and the risk of incident sudden cardiac death among adults with and without diabetes: over 15 years of follow-up in The Tehran Lipid and Glucose Study
Source: Diabetol Metab Syndr. 2021 Jul 28;13:82. doi: 10.1186/s13098-021-00701-z (PMC8320203; doi:10.1186/s13098-021-00701-z)
Supplement: Supplementary file 5 — Additional file 5: Table S3. Multivariable hazard ratios (HR) and 95% confidence intervals (CI) of different anthropometric indices (as categorical variables) for incident sudden cardiac death (SCD) among female participants with diabetes: Tehran Lipid and Glucose Study, Iran, 1999-2018. [file 13098_2021_701_MOESM5_ESM.docx]

| **Supplementary Table 3. Multivariable hazard ratios (HR) and 95% confidence intervals (CI) of different anthropometric indices (as categorical variables) for incident sudden cardiac death (SCD) among female participants with diabetes: Tehran Lipid and Glucose Study, Iran, 1999-2018.** | | | | | | |
| --- | --- | --- | --- | --- | --- | --- |
|  | **Quartile Range** | **E/N** | **Model 1** | | **Model 2** | |
|  |  |  | **HR (95% CI)** | **p-value** | **HR (95% CI)** | **p-value** |
| **BMI** |  | |  |  |  |  |
| **First Quartile** | < 25.9 Kg/m^2^ | 12/132 | **Reference** |  | **Reference** |  |
| **Second Quartile** | 25.9-28.5 Kg/m^2^ | 9/152 | 0.63 (0.27-1.50) | 0.296 | 0.53 (0.22-1.27) | 0.155 |
| **Third Quartile** | 28.5-31.6 Kg/m^2^ | 10/172 | 0.68 (0.29-1.58) | 0.370 | 0.67 (0.28-1.59) | 0.360 |
| **Fourth Quartile** | 31.6 Kg/m^2^ ≤ | 8/226 | 0.43 (0.17-1.05) | 0.064 | 0.37 (0.15-0.92) | 0.033 |
| **P-value for trend** |  |  |  | 0.084 |  | 0.059 |
| **WC** |  | |  |  |  |  |
| **First Quartile** | < 91 cm | 12/191 | **Reference** |  | **Reference** |  |
| **Second Quartile** | 91-98 cm | 7/168 | 0.59 (0.23-1.50) | 0.267 | 0.44 (0.17-1.14) | 0.092 |
| **Third Quartile** | 98-105 cm | 11/143 | 1.15 (0.50-2.60) | 0.744 | 0.92 (0.40-2.15) | 0.855 |
| **Fourth Quartile** | 105 cm ≤ | 9/180 | 0.64 (0.27-1.53) | 0.315 | 0.51 (0.21-1.24) | 0.137 |
| **P-value for trend** |  |  |  | 0.581 |  | 0.345 |
| **WHR** |  | |  |  |  |  |
| **First Quartile** | < 0.90 | 9/254 | **Reference** |  | **Reference** |  |
| **Second Quartile** | 0.90-0.95 | 7/193 | 0.80 (0.30-2.15) | 0.656 | 0.70 (0.26-1.91) | 0.488 |
| **Third Quartile** | 0.95-1.01 | 13/152 | 1.73 (0.72-4.14) | 0.222 | 1.42 (0.58-3.46) | 0.437 |
| **Fourth Quartile** | 1.01 ≤ | 10/83 | **2.31 (0.91-5.86)** | **0.077** | 1.96 (0.77-5.00) | 0.160 |
| **P-value for trend** |  |  |  | **0.032** |  | **0.083** |
| **WHtR** |  | |  |  |  |  |
| **First Quartile** | < 0.55 | 5/103 | **Reference** |  | **Reference** |  |
| **Second Quartile** | 0.55-0.60 | 10/138 | 1.23 (0.42-3.61) | 0.707 | 1.15 (0.39-3.40) | 0.807 |
| **Third Quartile** | 0.60-0.65 | 10/189 | 0.89 (0.30-2.63) | 0.836 | 0.76 (0.26-2.25) | 0.617 |
| **Fourth Quartile** | 0.65 ≤ | 14/252 | 0.78 (0.28-2.21) | 0.645 | 0.68 (0.24-1.95) | 0.479 |
| **P-value for trend** |  |  |  | 0.384 |  | 0.262 |
| **HC** |  | |  |  |  |  |
| **First Quartile** | < 97 cm | 9/113 | **Reference** |  | **Reference** |  |
| **Second Quartile** | 97-103 cm | 14/153 | 1.39 (0.60-3.22) | 0.446 | 1.50 (0.64-3.51) | 0.351 |
| **Third Quartile** | 103-109 cm | 7/167 | 0.59 (0.22-1.58) | 0.291 | 0.54 (0.20-1.47) | 0.228 |
| **Fourth Quartile** | 109 cm ≤ | 9/249 | 0.57 (0.23-1.46) | 0.242 | **0.64 (0.25-1.67)** | **0.364** |
| **P-value for trend** |  |  |  | **0.073** |  | **0.102** |
| E: event; N: number; BMI: body mass index; WC: waist circumference; WHR: waist-to-hip ratio; WHtR: waist-to-height ratio; HC: hip circumference; CVD: cardiovascular disease.  Model 1 was adjusted for age and sex. Model 2 was further adjusted for current smoking, education level, positive history of cardiovascular disease, family history of premature cardiovascular disease, hypertension, hypercholesterolemia, and FPG level. | | | | | | |
