# Supplementary material for: Anthropometric indices and the risk of incident sudden cardiac death among adults with and without diabetes: over 15 years of follow-up in The Tehran Lipid and Glucose Study
Source: Diabetol Metab Syndr. 2021 Jul 28;13:82. doi: 10.1186/s13098-021-00701-z (PMC8320203; doi:10.1186/s13098-021-00701-z)
Supplement: Supplementary file 6 — Additional file 6: Table S4. Multivariable hazard ratios (HR) and 95% confidence intervals (CI) of different anthropometric indices (as categorical variables) for incident sudden cardiac death (SCD) among male participants with diabetes: Tehran Lipid and Glucose Study, Iran, 1999-2018. [file 13098_2021_701_MOESM6_ESM.docx]

| **Supplementary Table 4. Multivariable hazard ratios (HR) and 95% confidence intervals (CI) of different anthropometric indices (as categorical variables) for incident sudden cardiac death (SCD) among male participants with diabetes: Tehran Lipid and Glucose Study, Iran, 1999-2018.** | | | | | | |
| --- | --- | --- | --- | --- | --- | --- |
|  | **Quartile Range** | **E/N** | **Model 1** | | **Model 2** | |
|  |  |  | **HR (95% CI)** | **p-value** | **HR (95% CI)** | **p-value** |
| **BMI** |  | |  |  |  |  |
| **First Quartile** | < 25.9 Kg/m^2^ | 14/165 | **Reference** |  | **Reference** |  |
| **Second Quartile** | 25.9-28.5 Kg/m^2^ | 16/144 | 1.15 (0.56-2.37) | 0.703 | 1.30 (0.61-2.76) | 0.497 |
| **Third Quartile** | 28.5-31.6 Kg/m^2^ | 7/125 | 0.61 (0.25-1.53) | 0.297 | 0.64 (0.25-1.63) | 0.350 |
| **Fourth Quartile** | 31.6 Kg/m^2^ ≤ | 10/69 | 1.67 (0.73-3.80) | 0.222 | 1.25 (0.51-3.07) | 0.633 |
| **P-value for trend** |  |  |  | 0.641 |  | 0.857 |
| **WC** |  | |  |  |  |  |
| **First Quartile** | < 91 cm | 11/151 | **Reference** |  | **Reference** |  |
| **Second Quartile** | 91-98 cm | 11/119 | 1.16 (0.50-2.67) | 0.729 | 1.12 (0.47-2.68) | 0.793 |
| **Third Quartile** | 98-105 cm | 15/136 | 1.21 (0.55-2.63) | 0.639 | 1.32 (0.59-3.00) | 0.498 |
| **Fourth Quartile** | 105 cm ≤ | 10/97 | 1.21 (0.51-2.86) | 0.658 | 0.96 (0.38-2.39) | 0.924 |
| **P-value for trend** |  |  |  | 0.635 |  | 0.913 |
| **WHR** |  | |  |  |  |  |
| **First Quartile** | < 0.90 | 2/45 | **Reference** |  | **Reference** |  |
| **Second Quartile** | 0.90-0.95 | 5/105 | 1.09 (0.21-5.63) | 0.917 | 0.92 (0.18-4.82) | 0.925 |
| **Third Quartile** | 0.95-1.01 | 18/181 | 2.22 (0.52-9.60) | 0.284 | 1.80 (0.41-7.98) | 0.438 |
| **Fourth Quartile** | 1.01 ≤ | 22/172 | **2.61 (0.61-11.1)** | **0.194** | 1.86 (0.42-8.14) | 0.410 |
| **P-value for trend** |  |  |  | **0.044** |  | **0.180** |
| **WHtR** |  | |  |  |  |  |
| **First Quartile** | < 0.55 | 13/194 | **Reference** |  | **Reference** |  |
| **Second Quartile** | 0.55-0.60 | 18/159 | 1.40 (0.68-2.87) | 0.358 | 1.34 (0.64-2.79) | 0.440 |
| **Third Quartile** | 0.60-0.66 | 9/108 | 0.98 (0.42-2.30) | 0.966 | 0.96 (0.40-2.29) | 0.919 |
| **Fourth Quartile** | 0.66 ≤ | 7/42 | 2.45 (0.98-6.14) | 0.056 | 1.50 (0.52-4.32) | 0.455 |
| **P-value for trend** |  |  |  | 0.236 |  | 0.699 |
| **HC** |  | |  |  |  |  |
| **First Quartile** | < 97 cm | 26/230 | **Reference** |  | **Reference** |  |
| **Second Quartile** | 97-103 cm | 12/149 | 0.70 (0.35-1.39) | 0.312 | 0.92 (0.45-1.88) | 0.816 |
| **Third Quartile** | 103-109 cm | 6/88 | 0.56 (0.23-1.37) | 0.203 | 0.67 (0.27-1.66) | 0.382 |
| **Fourth Quartile** | 109 cm ≤ | 3/36 | 0.60 (0.18-1.98) | 0.400 | **0.30 (0.08-1.13)** | **0.075** |
| **P-value for trend** |  |  |  | **0.149** |  | **0.062** |
| E: event; N: number; BMI: body mass index; WC: waist circumference; WHR: waist-to-hip ratio; WHtR: waist-to-height ratio; HC: hip circumference; CVD: cardiovascular disease.  Model 1 was adjusted for age and sex. Model 2 was further adjusted for current smoking, education level, positive history of cardiovascular disease, family history of premature cardiovascular disease, hypertension, hypercholesterolemia, and FPG level. | | | | | | |
